# Association of passive and active smoking with self-rated health and life satisfaction in Iranian children and adolescents: the CASPIAN IV study

**DOI:** 10.1136/bmjopen-2016-012694

**Published:** 2017-02-14

**Authors:** Ramin Heshmat, Mostafa Qorbani, Saeid Safiri, Amir Eslami-Shahr Babaki, Nassim Matin, Nazgol Motamed-Gorji, Mohammad-Esmaeil Motlagh, Shirin Djalalinia, Gelayol Ardalan, Morteza Mansourian, Hamid Asayesh, Roya Kelishadi

**Affiliations:** 1 Chronic Diseases Research Center, Endocrinology and Metabolism Population Sciences Institute, Tehran University of Medical Sciences, Tehran, Iran; 2 Non-Communicable Diseases Research Center, Alborz University of Medical Sciences, Karaj, Iran; 3 Endocrinology and Metabolism Research Center, Endocrinology and Metabolism Clinical Sciences Institute, Tehran University of Medical Sciences, Tehran, Iran; 4 Managerial Epidemiology Research Center, Department of Public Health, School of Nursing and Midwifery, Maragheh University of Medical Sciences, Maragheh, Iran; 5 Department of Pediatrics, Ahvaz Jundishapour University of Medical Sciences, Ahvaz, Iran; 6 Development of Research and Technology Center, Deputy of Research and Technology, Ministry of Health and Medical Education, Tehran, Iran; 7 Child Growth and Development Research Center, Research Institute for Primordial Prevention of Non-Communicable Disease, Isfahan University of Medical Sciences, Isfahan, Iran; 8 Health Management and Economics Research Center, Iran University of Medical Sciences, Tehran, Iran; 9 Department of Medical Emergencies, Qom University of Medical Sciences, Qom, Iran

**Keywords:** STATISTICS & RESEARCH METHODS

## Abstract

**Objective:**

To assess the joint association of passive and active smoking with self-rated health and life satisfaction among Iranian children and adolescents.

**Methods:**

Using a multistage random cluster sampling method, a representative sample of 14 880 school students were selected from urban and rural areas of 30 provinces of Iran. Data were gathered using a questionnaire, a weight scale and metre. Participants were classified into four groups based on their smoking patterns: ‘non-smoker’, ‘only active smoker’, ‘only passive smoker’ and ‘active and passive smoker’. Life satisfaction (LS) and self-rated health (SRH) were assessed by self-administered validated questionnaires based on the WHO-Global School-based student Health Survey (WHO-GSHS). Data were analysed using a t-test, χ^2^ test and multiple logistic regression.

**Results:**

A total of 13 486 individuals (6640 girls and 6846 boys) out of 14 880 invited participated in the study (response rate 90.6%). LS and good SRH showed linearly negative associations with smoking status in both sexes. The proportions of LS and SRH categories were significantly different among all subsets of smoking status. Those classified as ‘non-smokers’ had the highest proportions of LS and good SRH, followed by ‘only passive smokers’ and ‘only active smokers’, while those with ‘active and passive smoking’ had the lowest proportions of LS and good SRH. In a multivariate model, students in the ‘active and passive smoking’ group had lower odds of LS (OR 0.43; 95% CI 0.32 to 0.57) and good SRH (OR 0.51; 95% CI 0.38 to 0.68) than those in the ‘non-smoker’ group. Students in the ‘only passive smoker’ group also had lower odds of LS (OR 0.75; 95% CI 0.67 to 0.83) and good SRH (OR 0.72; 95% CI 0.66 to 0.80) compared with the ‘non-smoker’ group.

**Conclusions:**

Adolescents with different smoking habits and exposure patterns have poorer SRH and LS than non-smokers. Both active and passive smoking status could affect LS and SRH in adolescents. Therefore, smoking prevention strategies should be considered as a health priority in school health services for promoting psychological well-being in children and adolescents.

**Strengths and limitations of this study**

- The main strength of the present study was its comprehensive approach in a large national representative sample of Iranian children and adolescents. We could not find a study with a similar design with which we could compare our results. It was designed and conducted based on standardised protocols of the WHO-Global School-based student Health Survey.
- A limitation of the study was its cross-sectional nature, which does not allow assessment of a causal relationship between smoking status and self-rated health and life satisfaction.
- Although the sample size was large, there were not enough students in the ‘only active smoker’ and ‘active and passive smoker’ groups, so it might introduce bias to the regression model.

## Introduction

Self-rated health (SRH) is a subjective way of measuring the health status of a society. Evaluating this concept is possible by simply asking individuals to rate their general health status as good or poor. The significance of SRH arises from the fact that it has been considered as a predictor of morbidity, mortality, healthcare attendance and health-comprising behaviours in children and adults.^1^ Its association with mortality has been mostly discussed in adults.^2^ ^3^ In children and adolescents, SRH has been suggested to be strongly associated with health outcomes other than mortality, including psychological and social functionality, health complaints and health service attendance.^4^ ^5^

Life satisfaction (LS) is another subjective perception of general well-being which is influenced by the overall personal, social and psychological aspects of a person's health.^6^ LS in children and adolescents is highly associated with the degree to which they feel control over their environment.^7–9^ Similar to SRH, poor LS in children and adolescents is associated with adverse health outcomes such as violence and low psychosocial functionality.^10^ ^11^ Lack of experience and incomplete problem solving skills in children and adolescents make them vulnerable to small changes in their surroundings, which could inevitably reduce their LS.^11^ ^12^

Because of the significance of both SRH and LS in predicting and influencing the health outcomes of children and adolescents, it seems crucial to identify determinants that affect SRH and LS in this population. Many adolescent studies have investigated the role of various factors in influencing SRH and LS—namely, gender, socioeconomic status (SES), income, school performance, weight status, body image, physical activity (PA) and screen time (ST).^4^ ^13–19^ Tobacco use is one of the social behavioural determinants that has been investigated in this regard.

Prior studies have investigated the physical and medical consequences of tobacco smoking. The long-term effects of smoking significantly increase the risk of cardiovascular diseases and their risk factors, cancers, morbidity, mortality and poor quality of life.^13–15^ Exposure to smoking (either active or passive) during adolescence and childhood (when individuals might not be aware of the consequences) inevitably increases the duration of exposure to tobacco and carcinogens, nicotine dependency, lower cessation rates and heavier smoking.^20–22^ The effects of smoking on LS and SRH have also been investigated in previous studies. Due to a lack of appropriate coping strategies and poor social adaptation, it is not uncommon for individuals to respond to these changes by engaging in high-risk behaviours such as smoking cigarettes or even substance abuse. The opposite relationship is also plausible, in which smoking or other risky behaviours result in decreased LS or SRH.^23–25^ Previous studies have therefore suggested smoking tobacco to be associated with lower LS (which could even resume in adulthood)^26^ ^27^ and poor SRH.^21^ ^28–30^

In Iran the prevalence of overall current smoking (daily and occasional smoking) was reported as approximately 14% in a previous large sample of Iranian students aged 11–18 years in 2003–2004.^31^ A previous study of the CASPIAN IV survey on the same study population as the present study reported a prevalence of 41.1% passive smokers and 2.6% current smokers (daily smoking).^32^

Most previous studies have identified the association of smoking exposure with physical illnesses, whereas limited evidence exists on the association of smoking with psychological illnesses such as LS and SRH. Furthermore, most of the studies on the association of tobacco and LS/SRH have been conducted in Western populations; evidence regarding the effects of children and adolescent tobacco usage on LS and SRH in Iran appear to be scarce. Therefore, the current study aims to assess the joint association of active and passive smoking with LS and SRH among Iranian children and adolescents.

## Methods

### Participants and study design

This study is part of the fourth survey of the Childhood and Adolescence Surveillance and Prevention of Adult Non-communicable disease (CASPIAN IV) study, an Iranian national study in which 14 880 children and adolescents aged 6–18 years from elementary, intermediate and high schools in rural and urban areas were surveyed in 2011–2012. Multistage cluster sampling methods were used in 30 provinces. Methodological details have been previously described elsewhere.^33^

Initially, eligible schools were stratified according to the information bank of the Ministry of Health and Medical Education and random selection was then performed on them. The sample size was estimated to be 480 students in each province, thus 48 clusters of 10 students in each province and an equal number of their parents (with total population of 14 880 students) were selected. Two questionnaires validated and designed based on the WHO Global School-based Student Health Survey (WHO-GSHS)^34^ were given to the subjects and one of their parents to gather demographic data and variables of interest. These questionnaires had previously undergone forward translation to Farsi language by health professionals, according to the WHO process of translation and adaptation of instruments.^33^

All physical determinants were evaluated by calibrated equipment and professional trained health workers following standard protocols. Weight was estimated to the nearest 100 g (0.1 kg) on a scale placed on the ground and subjects were weighed shoeless and wearing light clothing. Height was measured, again without shoes, to the nearest 0.1 cm.

### Definition of terms

Dependent (outcome) variables of the current study include SRH and LS, which were validated in the previous study in Iran.^34^ SRH was assessed by a single question, “How would you describe your general state of health?”, categorised as ‘good’ or ‘poor’. LS was measured using a 10-point scale from 1 (very dissatisfied) to 10 (very satisfied). Scores >6 were further classified as satisfied and used in analysis.

Body image was assessed by a single item question, “How is your body size?”, and further categorised to underweight, normal and obese. Body mass index (BMI) was assessed using weight (in kg) divided by height (in m^2^). The WHO standard curves were used to categorise BMI into four groups of underweight (BMI less than 5th percentile for age and gender), normal weight (BMI between 5th and 85th percentiles for age and gender), overweight (BMI between 85th and 95th percentiles for age and gender) and obese (BMI greater than the 95th percentile for age and gender).

For evaluation of the SES score, the Principal Component Analysis (PCA) method was used. Students were classified into low, moderate and high SES based on parent education and job, type of school (private or governmental) and family assets (private car and computer).

ST was assessed through two determinants: time spent watching TV (TV time) and time spent working on a computer (computer/PC time). Subsequently, total ST was cumulatively computed and categorised as high (>2 hours per day) and low (≤2 hours per day) watching TV or computer work.

PA was evaluated by the question: “During the past week, on how many days were you physically active for overall 30 minutes per day?”, to which responses ranged from 0 to 7 days. PA was therefore categorised into three groups based on the number of days with at least 30 min of PA both in school and out of school (<2 days per week, 2–4 days per week and >4 days a week were classified as mild, moderate and vigorous PA, respectively).

Hours of sleep per week were also divided into <5, 5–8 and >8 hours. Two questions were allocated to depression and anxiety: “During the past 12 months, did you ever feel so sad or hopeless almost every day for 2 weeks or more in a row that you stopped doing your usual daily activities?” (response options: no and yes) and “During the past 6 months, how often did you experience anxiety so that you could not perform your daily activity?” (response options: almost every day (considered as yes), more than once a week (considered as yes), almost every week (considered as yes), almost every month (considered as no) and rarely or never (considered as no)).

For the purpose of this study, smoking (either active or passive) was examined in detail, with students being defined as either active or passive smokers. Those who reported use of tobacco products (eg, cigarette, pipe, hookah) every day (current smokers) were defined as active smokers and those who reported that people smoked tobacco products in their presence were considered as passive smokers. Thereafter, participants were classified into four subgroups of a combined determinant based on their smoking patterns: ‘non-smoker’, ‘only active smoker’, ‘only passive smoker’ and ‘active and passive smoker’.

### Statistical analysis

Categorical variables are presented as percentage and 95% CI. Continuous variables are reported with 95% CI. The mean age between the sexes was compared using a t-test; χ^2^ was used to assess the association of smoking status with LS and SRH. Three different logistic regression models were used to evaluate the association of SRH and LS with smoking. Model I represents the crude association; in model II, variables were adjusted for age, sex and region; and model III represents further adjustment for ST, PA, SES, family size, depression, sleeping hours, anxiety, body image and BMI. Non-smokers are defined as the reference group in each model. Before running models, outliers were excluded and collinearity between variables was tested and, after conﬁrming the lack of collinearity, the models were run. Goodness of fit (GOF) of the model was assessed using the Hosmer–Lemeshow test. Data were analysed using the survey data analysis method and analysis was weighted based on the population of each province. Statistical analysis was performed using Stata Statistical Software: Release V.12. (Stata Corp 2011, College Station, Texas, USA). p Values <0.05 were considered statistically significant.

## Results

A total of 13 486 individuals (6640 girls and 6846 boys) out of 14 880 participated in the study (response rate 90.6%). Mean values for age, BMI and LS score with corresponding CIs are shown in table 1 and showed no significant differences between the sexes. Also, the proportion of those living in urban areas, those within each category of SES (low, intermediate or high), those with different weekly sleep hours, those with good SRH (as defined earlier) and those with passive cigarette exposure were not significantly different between male and female participants (p<0.05).

**Table 1:** Characteristics of participants according to sex: the CASPIAN IV study

Variable	Boys (n=6846)	Girls (n=6640)	Total (n=13 486)	p Value
Age (years)*	12.36 (12.12 to 12.61)	12.58 (12.34 to 12.82)	12.47 (12.30 to 12.65)	0.21
BMI (kg/m^2^)*	18.74 (18.54 to 18.94)	18.97 (18.77 to 19.18)	18.85 (18.71 to 19.00)	0.11
Living place†				
Urban	74.89 (71.73 to 77.80)	76.27 (73.14 to 79.13)	75.57 (73.26 to 77.74)	0.50
Rural	25.11 (22.20 to 28.27)	23.73 (20.87 to 26.86)	24.43 (22.26 to 26.74)	
Screen time† (hours)				
≤2	78.07 (76.6 to 79.48)	84.78 (83.61 to 85.88)	81.38 (80.43 to 82.29)	<0.001
>2	21.93 (20.52 to 23.40)	15.22 (14.12 to 16.39)	18.62 (17.71 to 19.57)	
Physical activity†				
Mild	28.75 (26.85 to 30.73)	39.61 (37.54 to 41.72)	34.11 (32.66 to 35.58)	
Moderate	35.62 (34.11 to 37.17)	37.97 (36.36 to 39.61)	36.78 (35.66 to 37.92)	<0.001
Severe	35.62 (33.66 to 37.63)	22.42 (20.83 to 24.09)	29.11 (27.8 to 30.46)	
SES†				
Low	33.18 (31.13 to 35.30)	33.77 (31.72 to 35.88)	33.47 (32.02 to 34.95)	
Intermediate	32.67 (31.13 to 34.25)	33.52 (32.02 to 35.06)	33.09 (32.01 to 34.19)	0.57
High	34.15 (31.81 to 36.57)	32.71 (30.45 to 35.04)	33.44 (31.82 to 35.10)	
Hours of sleep per week†				
<5	0.58 (0.40 to 0.82)	0.73 (0.54 to 0.98)	0.65 (0.52 to 0.82)	0.57
5–8	22.39 (20.92 to 23.93)	22.91 (21.47 to 24.42)	22.65 (21.59 to 23.74)	
>8	77.03 (75.47 to 78.53)	76.36 (74.81 to 77.84)	76.70 (75.59 to 77.78)	
Passive smoker†	44.07 (42.42 to 45.74)	43.66 (42.05 to 45.28)	43.87 (42.73 to 45.01)	0.73
Active smoker†	3.49 (2.91 to 4.18)	1.66 (1.32 to 2.08)	2.59 (2.24 to 2.99)	<0.001
Smoking status†				
Non-smoker	55.81 (54.16 to 57.45)	56.90 (55.27 to 58.51)	56.35 (55.21 to 57.48)	<0.001
Only passive smoker	40.70 (39.11 to 42.30)	41.45 (39.87 to 43.04)	41.06 (39.96 to 42.18)	
Only active smoker	1.01 (0.76 to 1.34)	0.24 (0.15 to 0.39)	0.63 (0.49 to 0.81)	
Active and passive smoker	2.48 (2.04 to 3.03)	1.42 (1.11 to 1.80)	1.96 (1.68 to 2.28)	
Depression†	19.17 (17.90 to 20.51)	22.88 (21.47 to 24.35)	20.99 (20.03 to 21.99)	<0.001
Anxiety†	21.63 (20.27 to 23.06)	28.87 (27.30 to 30.49)	25.20 (24.13 to 26.30)	<0.001
Good SRH†	80.51 (79.32 to 81.65)	79.40 (78.13 to 80.61)	79.96 (79.10 to 80.80)	0.20
LS score‡*	8.09 (8.02 to 8.17)	8.19 (8.11 to 8.27)	8.14 (8.09 to 8.20)	0.08
LS†	79.58 (78.22 to 80.88)	80.25 (78.91 to 81.54)	79.91 (78.95 to 80.84)	0.47

p<0.05 is significant.

*Mean (95% CI).

†Per cent (95% CI).

‡Range of LS score is 0–10.

BMI, body mass index; LS, life satisfaction; SES, socioeconomic status; SRH, self-rated health.

Of the factors with significant differences between the sexes, boys had a higher proportion with generalised obesity and active cigarette exposure, while girls had a higher proportion with depression and anxiety. In addition, the proportion with various categories of perceived body image (underweight, normal and overweight), ST, PA intensity (mild, moderate and severe) and smoking status differed between the two groups; overall, a slightly higher proportion of boys were underweight and had normal body image, high ST (>2 hours), severe PA and components of active cigarette exposure. All baseline characteristics are shown in table 1.

In table 2 the proportions of dichotomous values for LS (satisfied vs dissatisfied) and SRH (good vs bad) are compared between individuals in the various smoking groups; comparisons were performed among boys, girls and the whole study population, separately. All values are shown as percentages with corresponding CIs. The proportions of different LS and SRH categories were different among all the smoking status subsets in girls, boys and the whole study population. When comparing LS as defined by satisfied and dissatisfied, among different subsets of cigarette exposure, generally most boys and girls (and a majority of the whole study population) were classified as ‘satisfied’ (p<0.001). However, upon further examination, in general a slightly lower proportion of boys were ‘satisfied’. Also, proportionally more passive smokers were ‘satisfied’. Furthermore, those classified as ‘non-smokers’ had the highest proportion described as ‘satisfied’, followed by ‘only passive smokers’ and ‘only active smokers’, while those with ‘active and passive smoking’ had the lowest proportion described as ‘satisfied’. This trend persisted in all groups (boys, girls and the total population).

**Table 2:** Association of smoking status with life satisfaction (LS) and self-rated health (SRH) according to sex: the CASPIAN IV study

Sex	Smoking status	LS			SRH		
		Satisfied	Dissatisfied	p Value*	Good	Bad	p Value*
Boys	Passive smoker†						
	Yes	75.31 (73.40 to 77.13)	24.69 (22.87 to 26.60)	<0.001	76.31 (74.52 to 78.01)	23.69 (21.99 to 25.48)	<0.001
	No	83.00 (81.45 to 84.43)	17.02 (15.57 to 18.55)		83.86 (82.51 to 85.12)	16.14 (14.88 to 17.49)	
	Active smoker†						
	Yes	59.41 (52.93 to 65.58)	40.59 (34.42 to 47.07)	<0.001	65.13 (58.69 to 71.05)	34.87 (28.95 to 41.31)	<0.001
	No	80.0 (78.98 to 81.59)	19.6 (18.41 to 21.02)		81.07 (79.88 to 82.20)	18.93 (17.80 to 20.12)	
	Smoking status†						
	Non-smoker	83.11 (81.59 to 84.53)	16.89 (15.47 to 18.41)	<0.001	83.92 (82.55 to 85.19)	16.08 (14.81 to 17.45)	<0.001
	Only passive smoker	76.51 (74.60 to 78.31)	23.49 (21.69 to 25.40)		77.18 (75.39 to 78.87)	22.82 (21.13 to 24.61)	
	Only active smoker	68.12 (54.64 to 79.12)	31.88 (20.88 to 45.36)		72.46 (61.07 to 81.53)	27.54 (18.47 to 38.93)	
	Active and passive smoker	55.88 (48.39 to 63.12)	44.12 (36.88 to 51.61)		62.13 (54.35 to 69.33)	37.87 (30.67 to 45.65)	
Girls	Passive smoker†						
	Yes	76.22 (74.20 to 78.12)	23.78 (21.88 to 25.80)	<0.001	75.43 (73.59 to 77.19)	24.57 (22.81 to 26.41)	<0.001
	No	83.53 (82.03 to 84.93)	16.47 (15.07 to 17.97)		82.68 (81.17 to 84.09)	17.32 (15.91 to 18.83)	
	Active smoker†						
	Yes	60.91 (52.02 to 69.13)	39.09 (30.87 to 47.98)	<0.001	63.64 (54.05 to 72.25)	36.36 (27.75 to 45.95)	<0.001
	No	80.05 (79.24 to 81.86)	19.42 (18.14 to 20.76)		79.67 (78.39 to 80.88)	20.33 (19.12 to 21.61)	
	Smoking status†						
	Non-smoker	83.33 (81.83 to 84.74)	16.67 (15.26 to 18.17)	<0.001	82.41 (80.90 to 83.83)	17.59 (16.17 to 19.10)	<0.001
	Only passive smoker	76.82 (74.82 to 78.72)	23.18 (21.28 to 25.18)		75.91 (74.04 to 77.68)	24.09 (22.32 to 25.96)	
	Only active smoker	75.00 (49.16 to 90.30)	25.00 (9.70 to 50.84)		75.00 (49.27 to 90.26)	25.00 (9.74 to 50.73)	
	Active and passive smoker	58.51 (48.36 to 67.99)	41.49 (32.01 to 51.64)		61.70 (51.23 to 71.19)	38.30 (28.81 to 48.77)	
Total	Passive smoker†	75.76 (74.36 to 77.10)	24.24 (22.90 to 25.64)	<0.001	75.88 (74.60 to 77.12)	24.12 (22.88 to 25.40)	<0.001
	Active smoker†	59.89 (54.64 to 64.91)	40.11 (35.09 to 45.36)	<0.001	64.66 (59.35 to 69.62)	35.34 (30.38 to 40.65)	<0.001
	Smoking status†						
	Non-smoker	83.22 (82.16 to 84.23)	16.78 (15.77 to 17.84)	<0.001	83.17 (82.14 to 84.15)	16.83 (15.85 to 17.86)	<0.001
	Only passive smoker	76.66 (75.26 to 78.01)	23.34 (21.99 to 24.74)		76.55 (75.26 to 77.79)	23.45 (22.21 to 24.74)	
	Only active smoker	69.41 (57.61 to 79.12)	30.59 (20.88 to 42.39)		72.94 (62.72 to 81.20)	27.06 (18.80 to 37.28)	
	Active and passive smoker	56.82 (50.77 to 62.67)	43.18 (37.33 to 49.23)		61.98 (55.78 to 67.81)	38.02 (32.19 to 44.22)	

p<0.05 is significant.

*p Values are for χ^2^ test.

†Per cent (95% CI).

For SRH, most subjects in all the subsets of cigarette exposure fell into the ‘good’ category (p<0.001). Overall trends were similar to those of LS—that is, a slightly higher proportion of passive smokers had ‘good’ SRH compared with active smokers—in all groups. In combined cigarette exposure status, ‘non-smokers’ had the highest proportion of ‘good’ SRH, followed by ‘only passive smokers’ and ‘only active smokers’, while those with ‘active and passive smoking’ had the lowest proportion of ‘good’ SRH.

The results of the regression models are presented in table 3. All models suggested that those with any degree of cigarette exposure (compared with ‘non-smokers’) had decreased odds of having both ‘satisfied’ LS status and ‘good’ SRH status to some extent. The data for ‘only active smokers’ did not show a significant difference in the adjusted model (Model III). In the multivariate model, students in the ‘active and passive smoking’ group had lower odds of LS (OR 0.43; 95% CI 0.32 to 0.57) and good SRH (OR 0.51; 95% CI 0.38 to 0.68) compared with the ‘non-smoker’ group. Students in the ‘only passive smoker’ group also had lower odds of LS (OR 0.75; 95% CI 0.67 to 0.83)) and good SRH (OR 0.72; 95% CI 0.66 to 0.80) compared with ‘non-smokers’. The results of the GOF test showed that our models fit reasonably well.

**Table 3:** ORs (95% CI) for life satisfaction (LS) and self-rated health (SRH) across smoking status: the CASPIANIV study

Model	Smoking status	LS (satisfied/dissatisfied)	SRH (good/poor)
Model I†	Non-smoker	Reference	Reference
	Only passive smoker	0.66 (0.60 to 0.73)*	0.66 (0.60 to 0.72)*
	Only active smoker	0.46 (0.27 to 0.77)*	0.55 (0.34 to 0.88)*
	Passive and active smoker	0.27 (0.21 to 0.34)*	0.33 (0.25 to 0.43)*
	p-trend	<0.001	<0.001
Model II‡	Non-smoker	Reference	Reference
	Only passive smoker	0.68 (0.62 to 0.75)*	0.67 (0.61 to 0.74)*
	Only active smoker	0.65 (0.39 to 1.10)	0.70 (0.43 to 1.13)
	Passive and active smoker	0.37 (0.29 to 0.47)*	0.43 (0.32 to 0.56)*
	p-trend	<0.001	<0.001
	F-adjusted GOF (p value)	0.68 (0.79)	1.1 (0.29)
Model III§	Non-smoker	Reference	Reference
	Only passive smoker	0.75 (0.67 to 0.83)*	0.72 (0.66 to 0.80)*
	Only active smoker	0.93 (0.52 to 1.66)*	0.84 (0.48 to 1.47)
	Passive and active smoker	0.43 (0.32 to 0.57)*	0.51 (0.38 to 0.68)*
	p-trend	<0.001	<0.001
	F-adjusted GOF (p value)	0.44 (0.91)	1.43(0.17)

*p<0.05.

†Without adjusted (crude models).

‡Adjusted for age, sex and region.

§Additionally adjusted for screen time, physical activity, socioeconomic status, depression, sleeping hours, anxiety and body mass index.

GOF, goodness of fit.

**Figure 1:**
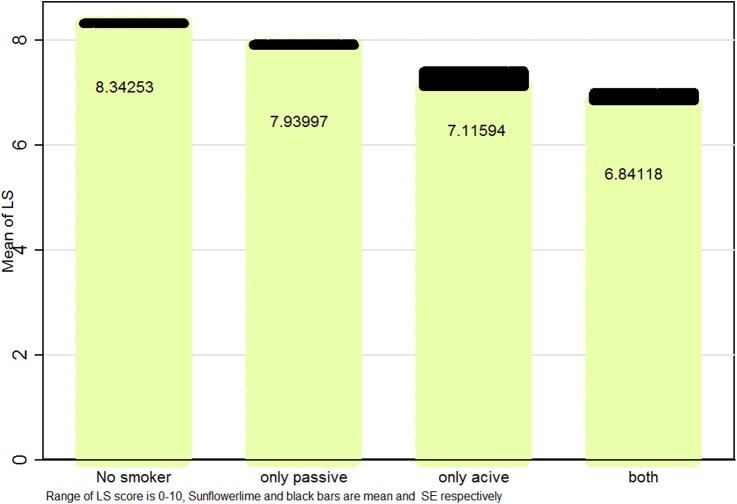
Mean (SE) life satisfaction (LS) score according to smoking status in Iranian male students: the CASPIAN IV study.

**Figure 2:**
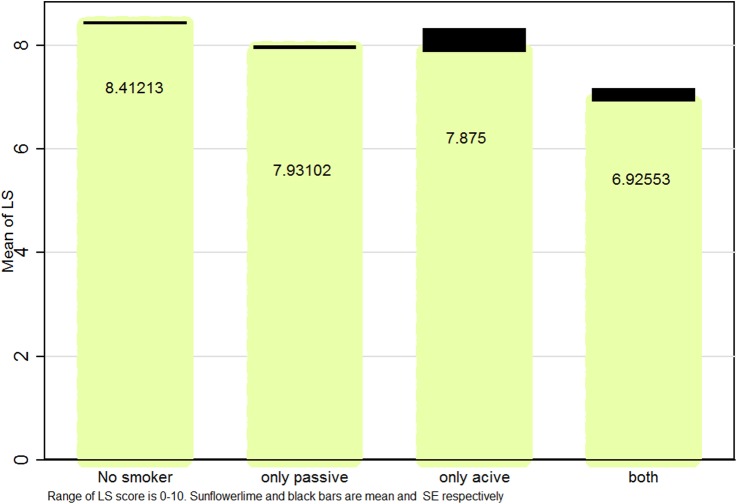
Mean (SE) life satisfaction (LS) score according to smoking status in Iranian female students: the CASPIAN IV study.

**Figure 3:**
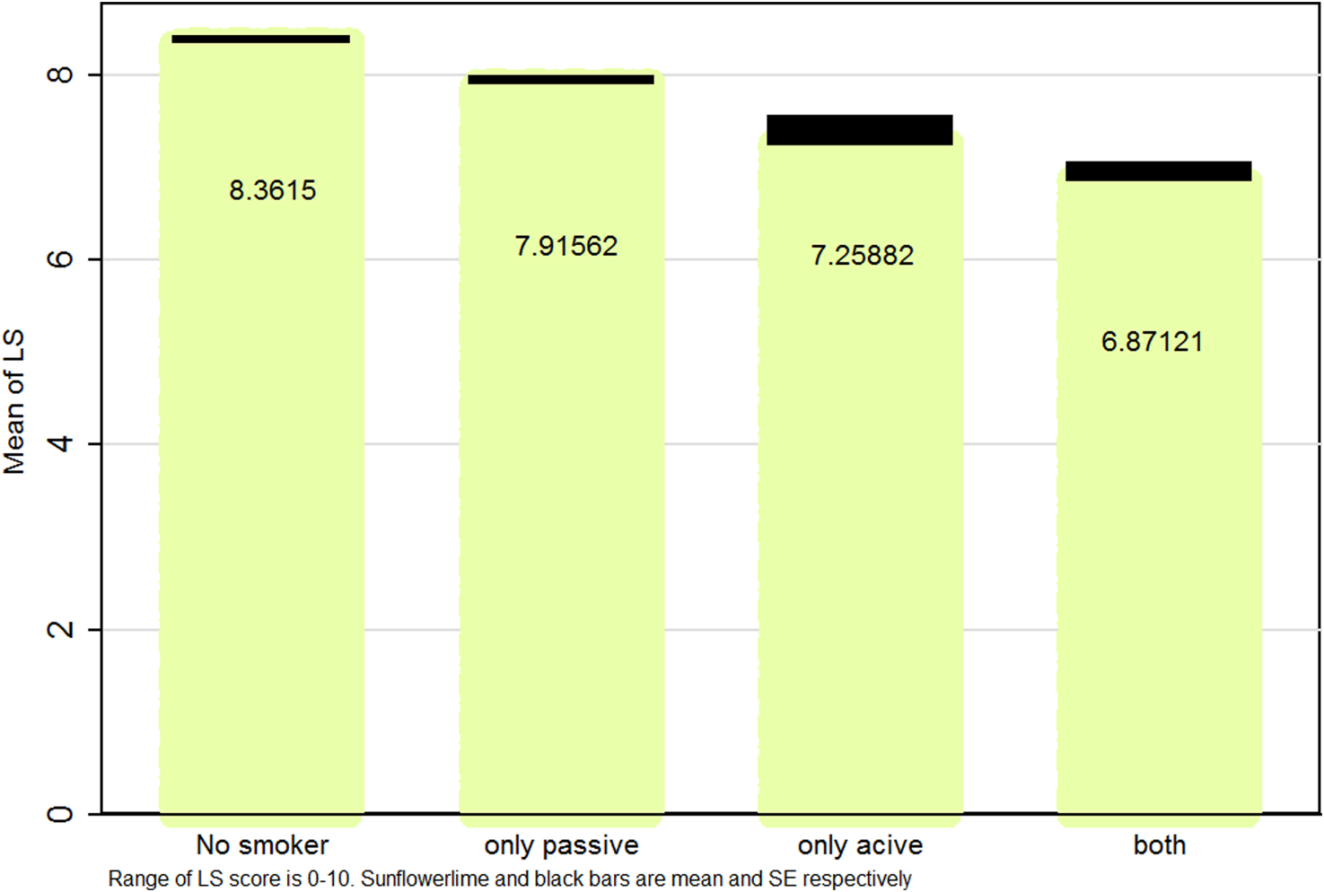
Mean (SE) life satisfaction (LS) score according to smoking status in Iranian students: the CASPIAN IV study.

Figures 1–3 illustrate mean and SEs of numerical values of LS based on smoking status in boys, girls and the whole study population, respectively. Overall, LS scores were higher in ‘non-smokers’, followed by ‘only passive smokers’, ‘only active smokers’ and ‘passive and active smokers’.

## Discussion

To the best of our knowledge, this is the first national study to investigate the joint association of passive and active smoking with SRH and LS in a large representative sample of Iranian children and adolescents. Our findings show that LS and SRH differed among individuals (girls, boys and the total study population) based on their smoking exposure. Theoretically, zero exposure (non-smoker) was associated with the highest absolute difference between SRH and LS figures in the various smoking categories. These differences gradually decreased as we moved from ‘non-smoker’ to ‘only passive smokers’, ‘only active smokers’ and ‘passive and active smokers’. Based on our crude regression model (Model I), increasing intensities of cigarette exposure significantly and substantially decreased the odds of having optimal LS and good SRH; being a ‘passive only smoker’ decreased these figures by almost one-third (33%) and being a ‘passive and active smoker’ lowered these values by two-thirds. When the model was adjusted for additional confounders (Models II), the deleterious effect of cigarette exposure was mitigated to some extent. This finding could be partially explained by gender differences. Adjusting the regression model for additional variables (Model III) negated the effects of smoking even further. Therefore, our data suggest that some confounders such as depression could have had a synergistic effect; however, the overall trend was persistent in both models with that of the crude model. Considering illustrations for quantitative LS values, one could see a role for gender in the LS score; both overall trends and boys display a tendency to have lower LS with increased tobacco exposure. However, in girls this trend is not as sharp as in boys. Overall, increased cigarette exposure is accompanied by lower LS, but the differences are more subtle. This finding may be explained by the higher prevalence of smoking in boys, resulting in a more significant impression.

In general, our results suggest that smoking behaviours have important influences on adolescents' self-perceived health and LS. In other words, children and adolescents who smoke or are exposed to smoking by other individuals have higher odds of being dissatisfied with their life and/or report their health status as bad. Some gender differences also emerge in this relationship, mainly stemming from differences in attitudes and attributed health behaviours to smoking in these age groups.

The findings of the present study appear to be consistent with previous studies in this regard. A similar CASPIAN IV study conducted in 2015 investigated the association of smoking with psychiatric distress and violence in children and adolescents. Although the study focused on specific health-related quality of life indicators other than LS and SRH, it was clearly shown that active and passive smoking are both associated with poorer psychiatric health and living environment conditions. This could in general be consistent with the results of the current study, since smoking, poor psychosocial status and violence could all synergistically result in a lower LS in adolescents,^32^ an association which has been suggested previously.^35^

In a 2001 study it was demonstrated that LS was significantly affected by substance abuse (including cigarette smoking, chewing tobacco, marijuana) in adolescents.^23^ Another study conducted in 2005 explored the interrelationships between adolescent smoking and specific social and personal outcomes (including LS) across four different countries. This study reported an overall lower frequency of smoking in adolescents who were satisfied with their life which, according to the study, could be justified and associated with self-concepts of individuals regarding their present and future status. In other words, the causality association between smoking status and LS could be reciprocal and bidirectional, meaning that an individual's perception of his/her situation could affect both LS and behaviours such as smoking.^26^

The association between smoking status and LS has been evaluated in adults as well. In a study conducted in 2015, subjective well-being and mindfulness of smoker and non-smoker adults were compared with each other. This study also showed that the level of satisfaction with life was higher in non-smokers, which is in agreement with our findings.^36^ The role of substances other than cigarettes has also been investigated. A study on the effects of hookah use in American adults led to similar findings, with the level of optimal subjective well-being being significantly lower in hookah users than in non-hookah users.^37^ The association between smoking status of adolescents and later adult LS has also been investigated. A cohort study published in 2007 showed that substance abuse in adolescence led to lower LS scores in young adulthood, and suggested that continuation of abuse may result in further intensification of the decrease.^38^ Therefore, the findings of the present study on the association between smoking and LS are consistent with previous research.

Our findings support previous results which emphasise that, in children and adolescents, health behaviours may have a strong influence on adolescents' SRH.^39^ ^40^ A 2004 Spanish study showed that adolescents with a daily smoking habit reported significantly higher frequencies of suboptimal SRH.^21^ Another study in 2007 achieved the same results.^41^ A study in 2012 reported that Chinese adolescents who smoked experienced lower SRH. This study suggested that, due to the observed association, SRH could be used as a sensitive indicator of health among seemingly healthy adolescents who smoke.^29^

Similar to the present research, most of the prior studies in adolescents have suggested that current daily smoking is a predictor of SRH. However, Vingilis *et al*^4^ investigated the role of occasional smoking and concluded that it had no significant effect on SRH of adolescents. Adult studies have also investigated the role of smoking in SRH. In a 2003 study on Hong Kong Chinese adults, current smokers reported poorer SRH than non-smokers or previous smokers, although the role of passive smoking was not evaluated.^42^

In most related studies, non-smokers had more positive SRH levels across all groups. On the other hand, there have been few studies that detected no association between smoking and poor SRH. In a 2009 study on predictors of SRH in adults, two groups of smokers and non-smokers were compared. This study reported that no association was observed between current smoking status and SRH. In spite of this, the study reported that, in smokers, the intensity of smoking was associated with poorer SRH.^43^ Accordingly, the probable reason for an adverse association of smoking with SRH and LS is related to the differences in attitudes that led to attributed health behaviours and health conception measures such as SRH and LS. That is to say, these studies believe that poor subjective health outcomes (namely SRH and LS) could be the consequences of internal or external stimulants which also lead to poor adjustment behaviours such as smoking; therefore there is no rationale for asserting an independent association between smoking and LS/SRH.^12^ ^44–46^ While it is clear that health behaviours are related to SRH, it is less clear if maintaining positive behaviours or improving them can protect SRH over time. Based on previous experiences, maintaining or increasing healthy lifestyle behaviours such as moderate PA or reducing smoking habits were associated with promotion of SRH.^45^ ^46^

Most of the studies investigating the role of smoking status in LS and SRH focused their assessment on a comparison of smoking versus non-smoking individuals or previous smokers (those who had quit smoking). In reviewing the literature, we rarely encountered studies that had compared the effects of active and passive (secondhand) smoking. However, one 2009 study in adults compared these states. In a sample of Japanese male and female full-time workers, it was shown that ‘not active smoker’ individuals who were occasionally exposed to secondhand smoking at work (passive smokers) had significantly increased levels of suboptimal SRH compared with non-smokers.^47^ A recent study in 2015 conducted on ‘not active smoker’ adults demonstrated that passive smoking was significantly associated with lower health-related quality of life indicators (ie, SRH).^48^ As mentioned previously, in searching the literature there were no reports comparing the effects of active and passive smoking on LS or SRH of adolescents. It should be noted that children and adolescents' lower control over their environment could result in higher degrees of exposure to secondhand smoking which, according to the present findings, could have various adverse effects on both objective and subjective well-being and health status.

Our findings underscore the role of active smoking in diminishing the subjective health of children and adolescents; furthermore, it reveals that even being passively exposed to smoking could effectively lower SRH and LS. Therefore, the adverse influence of passive smoking in children and adolescents should be highlighted more extensively.

To the best of our knowledge, the present study is the first Iranian research to investigate the joint association of active and passive smoking on SRH and LS in a population-based paediatric group. It is also the first study of this kind in the Middle East and North Africa (MENA) region. As already mentioned, many studies have noted the effects of smoking on different health outcomes in children and adolescents, but mostly indicated their effects on medical outcomes; health-related quality of life indicators such as SRH and LS have not been getting enough attention. Therefore, further studies are required to assess the association of passive and active smoking with LS and SRH, specifically in Middle-Eastern children and adolescents.

## Conclusions

The findings of the present study show that adolescents with different smoking habits or exposure patterns have lower SRH and LS compared with non-smokers. The results of the present study highlight the importance of smoking prevention strategies in children and adolescents. In addition, parents and other caregivers should be aware of the deleterious effects of passive smoking in children and adolescents. These strategies should be considered as a health priority in school health services, as well as macroplanning for promoting psychological well-being in children and adolescents.
